# Evaluating the Utility of Using Text Messages to Communicate With Patients During the COVID-19 Pandemic

**DOI:** 10.5435/JAAOSGlobal-D-21-00042

**Published:** 2021-06-15

**Authors:** Kevin J. Campbell, Brenna E. Blackburn, Jill A. Erickson, Christopher E. Pelt, Lucas A. Anderson, Christopher L. Peters, Jeremy M. Gililland

**Affiliations:** From the Orthopedic & Sports Institute of the Fox Valley, Appleton, Wisconsin (Dr. Campbell), and the Department of Orthopedics, University of Utah, Salt Lake City, Utah (Dr. Campbell, Dr. Blackburn, Ms. Erickson, Dr. Pelt, Dr. Anderson, Dr. Peters, and Dr. Gililland).

## Abstract

**Introduction:**

We evaluated the use of text messages to communicate information to patients whose surgeries were postponed because of the COVID-19 restriction on elective surgeries. Our hypothesis was that text messaging would be an effective way to convey updates.

**Methods:**

In this observational study, 295 patients received text messaging alerts. Eligibility included patients who had their surgery postponed and had a cell phone that received text messages. Engagement rates were determined using embedded smart links. Patient survey responses were collected.

**Results:**

A total of 3,032 texts were delivered. Engagement rates averaged 90%. Survey responses (n = 111) demonstrated that 98.2% of patients liked the text messages and 95.5% said that they felt more connected to their care team; 91.9% of patients agreed that the text updates helped them avoid calling the office. Patients with higher pain levels reported more frustration with their surgery delay (5.3 versus 2.8 on 1 to 10 scale, *P* value < 0.01). More frustrated patients wished they received more text messages (24.4% versus 4.6%, *P* value = 0.04) and found the content less helpful (8.2 versus 9.2 on 1 to 10 scale, *P* value = 0.01).

**Conclusion:**

Text messaging updates are an efficient way to communicate with patients during the COVID-19 pandemic.

On March 16, 2020, the State Department of Health issued a 6-week ban on nonurgent surgeries in an attempt to preserve essential healthcare resources and prepare for COVID-19 surge conditions. This ban, along with the persistent presence of the virus within our community, brought many changes to our academic joint arthroplasty practice, including the widespread cancellation of elective surgery and the rapid adoption of telehealth services. A particular challenge for our group was keeping a growing cohort of patients, whose elective joint arthroplasty surgeries were either delayed or canceled because of this ban, informed and updated because our office navigated the dynamic conditions of the pandemic.

In our group, which consists of four high-volume hip and knee arthroplasty specialists, the surgical dates for 295 patients were either directly or indirectly affected by the 6-week nonurgent surgery ban at its outset. It became apparent to us that we needed a way to keep them engaged with our office for a number of reasons. First, understanding that our patients were facing many unknowns regarding their surgery, we wanted to keep them informed about scheduling updates (or lack thereof) and help manage their expectations in a thoughtful and timely manner. Second, it was important to ensure that our patients did not experience an additional decline in their health status or mobility because they awaited surgery. We wanted to reiterate the importance of healthy habits, such as smoking cessation, weight management, and management of comorbid medical conditions. Third, we wanted to distribute reputable information to them about COVID-19 because it relates to their orthopaedic condition (eg, American Academy of Hip and Knee Surgeons [AAHKS] patient education materials). Fourth, we wanted to maintain the patient-physician relationship so that they would continue to rely on us for their orthopaedic care when elective surgeries resumed. Finally, we wanted to convey a sense of empathy although we were not able to accommodate in-person clinical visits or connect with all of these patients individually on a regular basis. Ideally, we wanted to attain these goals without overburdening clinical staff and resources.

Just before the COVID-19 shutdown, we had started using a text messaging patient engagement program to help our patients prepare for and recover from surgery. Based on our conversations with patients, we found that traditional text messaging (short message service, SMS) is a convenient and simple way for them to receive information. Although the standard use case for the text program is the automation of perioperative communication through the automatic delivery of informative text messages, we adopted a novel use for it by sending text updates to our patients whose surgeries were either postponed or canceled because of COVID-19. The content and timing of the messages included a breadth of topics, such as relevant updates from our office, information for nonsurgical arthritis care, patient education materials from our department as well as the AAHKS (AAHKS Patient Education Portal), and personalized video messages from our attending surgeons, physician assistants, and physical therapists.

The purpose of this study was to evaluate the use of text messages to communicate updates and information to our patients whose surgeries had been postponed during the COVID-19 pandemic. Our hypothesis was that text messages during the COVID-19 pandemic would be regarded by our patients as an easy way to receive information and would not overly burden our staff or strain clinical resources.

## Methods

In this IRB exempt study, we prospectively sent text message updates to our patients whose elective surgeries were postponed because of the COVID-19 pandemic. A total of 338 patients were initially affected by the restriction. Of those, 295 (87% enrollment rate) signed up and consented to receive the text updates. The number of patients receiving the text alerts grew to approximately 350 throughout the study period because more cases were postponed. Text message updates commenced on April 3 and continued to May 27, at which time our institution transitioned to the yellow “low-risk” phase, and elective surgeries resumed. The text messaging service was provided by STREAMD.

During the 8-week study period, patients received 3,032 text updates, approximately 10 messages per patient. The content of the messages included relevant updates from our office, information for nonsurgical arthritis care, patient education material from our department as well as the AAHKS (AAHKS Patient Education Portal), and personalized video messages from our attending surgeons, physician assistants, and physical therapists. To measure engagement, text messages that included a web address were embedded with a “smart” URL link (Bitly) capable of tracking the number of times the patients clicked on the URL. Responses from patients into the system were not encouraged. An automatic reply directing patients to call the office with questions was programmed to respond to inbound messages from patients. After the final text was delivered, we collected survey responses (Appendix 1, http://links.lww.com/JG9/A143) to gauge patients' satisfaction with the text alerts. Overall responses were calculated and stratified by age, sex, pain level, and frustration level. Descriptive statistics, including chi-square and *t*-tests, were used for these comparisons.

## Results

A total of 3,032 texts were delivered (∼10 per patient). Nine patients unenrolled themselves in the text messaging service (one had manipulation under anesthesia during the study period, three had surgery at another facility that was offering elective surgery despite the directive during the study period, and five canceled and did not want to reschedule their surgery). Engagement rates averaged 90%. This calculation is based on the average percent viewership of the embedded “smart” URL links in the text messages. Overall, 111 patients completed the survey, totalling a response rate of 32% (111/350). The average age of respondents was 64.3 years (range = 19 to 86), with most being female (70.3%) (Table 1). Survey responses demonstrated that 98.2% of patients liked the text messages and 95.5% said that they felt more connected to their care team (Table 2); 91.9% of patients agreed that the text updates helped them avoid calling the office; and 85.6% of patients preferred text updates compared with e-mail, phone calls, or patient portal messages.

**Table 1:** Characteristics of Patients Who Completed the Survey

	N = 111
	Mean (Std)
Age	64.3 (12.5)
	N (%)
Sex	
Male	33 (29.7)
Female	78 (70.3)
Body part	
Hip	50 (45.1)
Knee	61 (55.0)

**Table 2:** Survey Results

	N = 111
	Mean (Std)
Pain (1-10)	5.8 (2.2)
Frustration (1-10)	4.4 (2.9)
How helpful was the content (1-10)	8.8 (1.7)
Liked receiving messages score	4.8 (0.4)
Text messages helped me feel connected score	4.6 (0.6)
	N (%)
Liked receiving messages	
Strongly agree	96 (86.5)
Agree	13 (11.7)
Neutral	2 (2.3)
Disagree	0
Strongly disagree	0
Frequency	
Wanted more	14 (12.6)
Just right	94 (84.7)
Too many	3 (2.7)
Text messages helped me feel connected	
Strongly agree	68 (61.3)
Agree	38 (34.2)
Neutral	5 (4.5)
Disagree	0
Strongly disagree	0
Helped avoid calling office	
Agree	102 (91.9)
Disagree	9 (8.1)
Would rather have received communication through:	
E-mail	6 (5.4)
MyChart	2 (1.8)
Letter in mail	0
Phone call	8 (7.2)
Preferred text messages	95 (85.6)
Content liked the most (check all that apply)	
Office surgery scheduling updates	101 (91.0)
Tips for at-home pain control	56 (50.5)
Video messages from healthcare team	52 (46.9)
Website links to local/state health department	15 (13.5)
Website links to educational content from AAHKS	23 (20.7)

AAHKS = American Academy of Hip and Knee Surgeons

A total of 84.7% of patients rated the frequency of the updates as just right; 91.0% of patients rated ‘scheduling updates' as the most valuable content. Patients with higher pain levels reported more frustration with their surgery delay (5.3 versus 2.8 on 1 to 10 scale, *P* value < 0.01). More frustrated patients wished they received more text messages (24.4% versus 4.6%, *P* value = 0.04) and found the content less helpful (8.2 versus 9.2 on 1 to 10 scale, *P* value = 0.01).

Of the seven text messages that included embedded destination URLs, the links with the highest patient engagement included video messages from attending surgeons (eg, Dr. #3 and Dr. #2 video message viewed 139% and 129%, respectively). The link with the third highest engagement (109%) directed patients to the AAHKS portal,^1^ which provides patients with peer-reviewed education resources written by surgeon members of the AAHKS Patient Education Committee. The lowest engagement rates were observed with links directed to the COVID-19 section of the State Department of Health website (24%) and a video message about home pain control modalities (34%). The engagement rates for the embedded links are shown in Table 3.

**Table 3:** Engagement rates for Embedded Links

Date	Link	Recipients	Clicks
April 6, 2020	Dr. #2 video message	89	117
April 8, 2020	Dr. #3 video message	49	69
April 9, 2020	PA-C video message	120	118
April 13, 2020	Department of Health|COVID-19 updates	277	68
April 15, 2020	Message from physical therapy	282	220
April 15, 2020	AAHKS|COVID-19 updates	300	341
May 9, 2020	Tips on pain control from PA-C	298	118

AAHKS = American Academy of Hip and Knee Surgeons

## Discussion

In this study, we sent our patients, whose surgery had been delayed because of the COVID-19 elective surgery restriction, text messages in an attempt to keep them informed, educated, and engaged to our office when we awaited the resumption of surgery. The most notable result from this study is that the patients found that text updates are a helpful and convenient way for them to receive general information from the office because their surgeries were postponed because of COVID-19. Because of the COVID-19 effect on our health system, surgical cancelations and postponements lasted for 8 weeks, and we still have not been able to return to pre-COVID surgical case levels. We learned rather quickly that our patients who were awaiting surgery desired some sort of connection to our office to help them cope with the anxiety regarding the uncertainty of COVID-19 and how it would affect their individual care.

To maintain communication with our patients whose surgery had been delayed, we searched internally for an appropriate communication platform. We considered e-mail, MyChart messages (EPIC Health System) and phone calls but realized there were specific limitations with each that made them unusable for this particular use case. For e-mail, it did not feel personal enough, and our experience has been that our patients are not reading their e-mails regularly. Our health system discouraged us from sending mass MyChart messages for the specific purpose in this study because they suspected it could be confusing to patients who also have internal medicine physicians and other specialists using the same MyChart portal as well. We also did not want to burden our patients by having them log into MyChart to read their messages. Finally, although phone calls seemed like the most personable way to connect, we have routinely had limited success getting our patients to answer their phone, given how ubiquitous robocalling has become. Our medical assistants get into a vicious cycle where they leave voicemails and then when patients return their calls, the assistants are already on another patient call, forcing the patient to leave a voicemail and wait for a callback. Moreover, calls tend to take quite a bit of time because patients start to have conversations with our staff about other unrelated topics. Ultimately, calling 300 + patients for each update we wanted to provide was not feasible for our staff.

Texting has increased in popularity year-after-year and has been adopted by every age group. It is reported that Gen X'ers (45 to 54 years) send and receive about 33 text messages per day and baby boomers (56 to 76 years) send and receive about 16 text messages per day.^2^ The benefits of texting a group of patients are the speed and the highly interactive nature of SMS. Texting is a native feature to every cell phone, and by mass texting, we were able to reach our patients quickly. In total, 90% of SMS marketing messages are opened within three minutes, and 99% are opened within 20 minutes.^3^ This is in stark contrast to e-mail message open rates, which have been reported to be 20%.^4^ Today, most patients are used to sending and receiving text messages, so by meeting them where they already are, we felt we were more likely to engage them. We did not feel this type of efficiency, and engagement could be found with any other communication channels.

In this study, we were not able to capture read rates for each individual text message, but we were able to gain insight into engagement because we tracked the click rates for the embedded URLs. We found click rates over 100% (meaning some patients clicked on the links more than one time) for the physician video messages and the AAHKS COVID-19 patient education portal. We were surprised by this finding, but feel strongly that physicians can have a strong calming effect for patients, especially during this time. These engagement rates suggest that text messaging is an acceptable and engaging communication platform. The average open rate for health and fitness e-mail campaigns is reported to be 21.48%, with click rates of only 2.69%.^5^ Although 97% of secure portal messages (eg, MyChart) are read by patients, it is reported that most hospitals (62%) see fewer than 25% of their patients activate their patient portal.^6^

Our study is not without limitations. This study was completed at a single academic center and may not be wholly representative of the patient population in other geographic areas. Moreover, this study is observational in nature, consisting of only a single cohort of patients who elected to receive text messages during the COVID-19 shutdown. We do not have a comparative cohort of patients who did not receive the messages. In addition, there is some selection bias at play because the only patients eligible were those who had access to a cell phone. In addition, we were unable to conceal patient allocation by blinding the participants. This has previously been reported to exaggerate the estimate of effect size of interventions.^7^ Finally, the patient self-reported data within our study are also subject to response bias, introspective ability, and the inherent limitations of rating scales and questionnaires.

## Conclusion

The restriction on elective surgeries presented our group with a unique challenge because we managed a growing list of patients whose surgeries were postponed or canceled. We sent text message alerts to our patients to keep them informed, educated, and engaged to our office because we navigated the pandemic. Patients reported high satisfaction and ease of use with this communication medium. We will continue to study the benefits of this technology going forward because we ramp up and return to a new sense of normalcy.
